# Unexpected features of breast cancer subtype

**DOI:** 10.1186/s12957-015-0665-8

**Published:** 2015-08-15

**Authors:** Ye-huan Liu, Ou-chen Wang, En-dong Chen, Ye-feng Cai, Chuan-meng Pan, Fan Yang, Xiao-hua Zhang

**Affiliations:** Department of Oncology, The First Affiliated Hospital of Wenzhou Medical University, South of Bai-xiang Street, Ou-hai District, 325000 Wenzhou, Zhejiang People’s Republic of China

## Abstract

**Background:**

Gene expression profiling of breast cancers identifies distinct molecular subtypes that affect prognosis. The aim of this study was to determine whether features of tumors especially the risks of lymph node (LN) metastases differ among molecular subtypes.

**Methods:**

Subtypes were classified by immunohistochemical surrogates as luminal A, luminalHer2−, luminalHer2+, TNBC, and HER-2+. Data were obtained from an established, registered database of patients with invasive breast cancer treated at our hospital between July 2012 and October 2014. A total of 929 tumors were classifiable into molecular subtypes.

**Results:**

The distribution of subtypes was luminal A (24.2 %), luminalHer2− (27.8 %), luminalHer2+ (9.1 %), TNBC (21.3 %), and HER-2+ (17.5 %). Marked differences in age, tumor size, extent of lymph node involvement, and grade were observed among subtypes. On univariate analysis, the LN positivity varied across subtypes with 33.6 % in luminal A, 40.3 % in luminalHer2−, 37.3 % in luminalHer2+, 37.6 % in TNBC, and 47.4 % in HER-2+ (*p* = 0.201). There was no significant difference in LN positivity among subtypes. On multivariable analysis, grade and tumor size were independent predictors of LN positivity.

**Conclusions:**

Predictors of LN metastases include higher grade and larger tumor size. Even though breast cancer subtype is not a statistically significant predictor of LN positivity, this information may still be useful in selecting the appropriate therapy in clinical practice.

## Background

Gene expression profiling based on 496 genes has identified distinct molecular subtypes of breast cancer that differ in prognosis. Studies have demonstrated that patients with HER-2+ (human epidermal growth factor receptor-2+) and TNBC (triple negative breast cancer) tumors have poorer survival compared with patients with luminal A and luminal B (luminalHer2− and luminalHer2+) tumors and that those with luminal A tumors have a better prognosis than those with luminal B tumors [1, 2]. Molecular subtype seems to be associated with the risk of local failure for patients treated with breast-conserving therapy and for those treated with mastectomy [3, 4]. However, little is known about whether the presenting characteristics especially the axillary lymph node status of breast cancer differ by molecular subtype. As we all know, nodal involvement carries important prognostic and therapeutic importance. Several predictors of lymph node metastasis have been described such as higher grade and larger tumor size [5, 6]. However, the impact of tumor subtype on axillary status has not been well established [1, 7–9].

Therefore, our purpose was to determine whether molecular subtype, as defined by estrogen receptor (ER), progesterone receptor (PR), Ki-67, and HER-2, correlates with presenting features of breast cancer, particularly to evaluate whether it is an independent predictor of axillary lymph node involvement on multivariable analysis.

## Methods

This study was performed with the approval of The First Affiliated Hospital of Wenzhou Medical University Review Board. Review of a prospectively maintained database of patients evaluated and treated for breast cancer from July 2012 to October 2014 was performed. Patients between the ages of 25 and 90 years with newly diagnosed stage I–III breast cancer were identified. Of these, preoperative systemic therapy was excluded.

Patients were categorized according to tumor phenotypic subtype using the presence or absence of tumor makers. Specifically, they were categorized into luminal A (ER+/PR+, HER2−, Ki67 < 14 % or PR ≥ 20 %), luminalHer2− (ER+/PR+, HER2−, Ki67 ≥ 14 % or PR < 20 %), luminalHer2+ (ER+ and HER-2+), TNBC (ER− and PR− and HER2−), and HER-2+ (ER− and PR− and HER-2+). The criteria are recommended according to the 13th St. Gallen International Breast Cancer Conference [10, 11]. ER and PR status was determined on the basis of immunohistochemical (IHC) staining. ER/PR was conceived to be positive if the percentage of nuclear-staining cancer cells is no less than 1 % [2, 9, 12]. Tumors were considered HER-2-positive only if they were scored 3+ by IHC or if they were HER-2-amplified (ratio ≥2.0) on the basis of fluorescence in situ hybridization (FISH) [13, 14]. Patient and tumor characteristics evaluated included age, tumor size, grade, molecular subtype, and nodal involvement. Nodal positivity was then evaluated based on the number of tumors involving lymph nodes. A positive node was defined as a lymph node containing any cancer cells by hematoxylin and eosin stain or cytokeratin positivity via IHC. This was divided into three groups: 0 node positive, 1–3 nodes positive, and ≥4 nodes positive. Characteristics that would result in nodal positivity were then evaluated and analyzed. These included patient age, tumor size, and breast cancer subtype.

The *χ*^2^ test was used for binary variables and analysis of variance for continuous variables to compare the distribution of clinicopathologic characteristics among the four subtypes. All percentages and statistical tests were based on available data. Multivariate logistic regression analysis was used to determine whether subtype was independently predictive of nodal involvement after controlling for age (continuous), tumor size (continuous), and tumor grade (3 vs. 2 vs. 1). Luminal A was the reference group. Patients with missing data were excluded from multivariate analysis. All statistical tests were two-sided, and a *p* value of <0.05 was considered significant. All statistical analyses were performed using SAS version 9.1 (SAS Institute, Cary, NC).

## Results

**Fig. 1:**
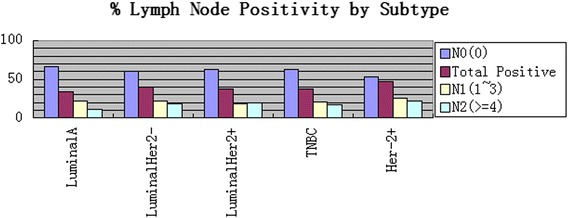
Number of total positive LN by subtype (*p* = 0.201). N0 vs. N1 vs. N2. More N0 in luminal A/TNBC, more N2 in luminalHer−, luminalHer+, and Her-2+

A total of 929 patients met the study criteria. Of these, 100 underwent breast-conserving surgery (BCS) and 829 were treated with mastectomy. The mean patient age was 52 (range, 25–90) years. Luminal A tumors were present in 24.2 %, luminalHer2− in 27.8 %, luminalHer2+ in 9.1 %, TNBC in 21.3 %, and HER-2+ in 17.5 %. Patient and tumor characteristics by subtype are summarized in Table 1. Among the four breast cancer subtypes, there were significant differences in the distribution of tumor size (all *p* = 0.002) and grade (all *p* < 0.0001). Luminal A tumors were smaller when compared to luminalHer2−, luminalHer2+, TNBC, and HER-2+ tumors (2.0 vs. 2.3, 2.3, 2.4, and 2.5; *p* = 0.001). Tumors overexpressing HER-2 (luminalHer2+ and HER-2+) and TNBC subtypes were more frequently in grade 3 and T3. HER-2+ tumors were more likely to have involvement of nodes. LN metastases were detected in 343 (39.1 %) patients. The LN positivity rate varied across subtypes with 73 of 217 (33.6 %) patients in luminal A, 96 of 238 (40.3 %) in luminalHer2−, 31 of 83 (37.3 %) in luminalHer2+, 70 of 186 (37.6 %) in TNBC, and 73 of 154 (47.4 %) in HER-2+. In addition, luminal A breast cancers were more frequently node-negative when compared to the others (66.4 vs. 59.7, 62.7, 62.4, and 52.6 %, respectively) and less frequently had four or more positive nodes (11.5 vs. 18.1,19.3,16.7 and 22.1 %, respectively) (Fig. 1). However, on univariate analysis, these data suggest that there was no significant difference in the incidence of nodal metastases among the four breast cancer subtypes (*p* = 0.201).

**Table 1:** Patient demographic and tumor data

Feature	Luminal A	luminalHer2−	luminalHer2+	TNBC	Her-2+	*p* value
Number, *n* (%)	225 (24.2)	258 (27.8)	85 (9.1)	198 (21.3)	163 (17.5)	
Age at diagnosis						
Mean ± SD	52.2 ± 12.1	52.3 ± 12.5	49.7 ± 10.1	52.1 ± 11.9	52.4 ± 10.4	0.433
Tumor size (cm)						
Mean ± SD	2.0 ± 1.1	2.3 ± 1.4	2.4 ± 1.4	2.5 ± 1.8	2.5 ± 1.5	**0.001**
Size distribution						
No. missing	7	4	1	4	3	
T1 (<2 cm)	119 (54.6)	101 (39.8)	39 (46.4)	70 (36.1)	55 (33.4)	**0.002**
T2 (2~5 cm)	95 (43.6)	144 (56.7)	41 (48.8)	117 (60.3)	96 (60.0)	
T3 (>5 cm)	4 (1.8)	9 (3.5)	4 (4.8)	7 (3.6)	9 (5.6)	
Grade, *n* (%)						
No. missing	19	25	6	15	11	
1	63 (30.6)	28 (12.0)	5 (6.3)	9 (4.9)	11 (7.2)	**<0.0001**
2	127 (61.7)	166 (71.2)	53 (67.1)	80 (43.7)	67 (44.1)	
3	16 (7.8)	39 (16.7)	21 (26.6)	94 (51.4)	74 (48.7)	
Node status						
No. missing	8	20	2	12	9	
Total positive	73 (33.6)	96 (40.3)	31 (37.3)	70 (37.6)	73 (47.4)	
N0 (0)	144 (66.4)	142 (59.7)	52 (62.7)	116 (62.4)	81 (52.6)	0.201
N1 (1~3)	48 (22.1)	53 (22.3)	15 (18.1)	39 (21.0)	39 (25.3)	
N2 (≥4)	25 (11.5)	43 (18.1)	16 (19.3)	31 (16.7)	34 (22.1)	

Bold values are statistically significant (*p* < 0.05)

*SD* standard deviation

On multivariate analysis, after controlling for tumor size, grade, and patient age, subtype was not a statistically significant predictor of nodal metastases (*p* = 0.227 in ≥1 positive LN and *p* = 0.561 in ≥4 positive LN; Table 2). When compared to the luminal A subtype, the odds ratio for LN positivity in HER-2+ was 1.2, with 95 % CI of 0.6–2.1, suggesting that HER-2+ has nodal involvement more frequently. However, none of the other subtypes was found to differ statistically significantly from the luminal A subtype in the increased risk of any nodal metastases. Furthermore, predictors of four or more positive nodes included size of the tumor of about 2~5 and >5 cm (odds ratio [OR] 2.4, 1.5–4.0, and OR 6.2, 1.5–26.4) (*p* = 0.001), and grade 2 or 3 tumors (OR 17.5, 2.4–130.5 and OR 22.9, 3.0–176.3) (*p* = 0.015). Age was not associated with an increased likelihood of positive lymph nodes. Larger size and higher grade were again found to be predictive of having one or more positive nodes. In addition, when evaluating the predictors of ≥4 positive nodes, tumors overexpressing HER-2 (luminalHer2+ and HER-2+) were more likely to have four or more nodes positive (OR 1.1, 0.5–2.7 and OR 1.4, 0.7–3.0) (Table 2).

**Table 2:** Multivariable logistic regression

Variable	≥1 Positive LN (*N* = 343)		≥4 Positive LN (*N* = 149)	
	OR (95 % CI)	*p* value	OR (95 % CI)	*p* value
Subtypeª		0.227		0.561
Luminal A	1		1	
luminalHer2−	0.7 (0.4–1.2)		1.0 (0.5–2.0)	
luminalHer2+	0.7 (0.4–1.4)		1.1 (0.5–2.7)	
TNBC	0.7 (0.4–1.2)		0.8 (0.4–1.7)	
Her-2+	1.2 (0.6–2.1)		1.4 (0.7–3.0)	
Tumor size (cm)		**<0.0001**		**0.001**
<2	1		1	
2~5	2.3 (1.6–3.4)		2.4 (1.5–4.0)	
>5	2.7 (0.7–10.2)		6.2 (1.5–26.4)	
Grade		**<0.0001**		**0.015**
1	1		1	
2	5.6 (2.8–11.2)		17.5 (2.4–130.5)	
3	4.5 (2.1–9.8)		22.9 (3.0–176.3)	
Age level		0.548		0.676
<50	1		1	
50–79	0.9 (0.6–1.3)		1.1 (0.7–1.8)	
>79	2.1 (0.3–13.1)		2.1 (0.3–13.8)	

Bold values are statistically significant (*p* < 0.05)

*LN* lymph node, *OR* odds ratio

ªSubtypes as defined as luminal A (ER+/PR+, HER2−, Ki67 < 14 % or PR ≥ 20 %), luminalHer2− (ER+/PR+, HER2−, Ki67 ≥ 14 % or PR < 20 %), luminalHer2+ (ER+ and HER-2+), TNBC (ER− and PR− and HER2−) and HER-2+ (ER− and PR− and HER-2+)

## Discussion

In this study, we found an unexpected result when comparing initial presenting characteristics of invasive breast cancer. On univariate analysis, factors associated with poor prognosis such as grade 3 and T3 were all far more frequent in tumors that overexpressed HER-2 and TNBC. On multivariate analysis, subtype was not a statistically significant predictor of any nodal involvement and high-volume nodal involvement (four or more positive lymph nodes). However, the HER-2+ subtype has nodal involvement more frequently when compared with the luminal A subtype.

Nodal status is an important factor associated with survival in breast cancer patients, and it is a major determinant factor in decision about therapy. Tumor size, tumor grade, tumor location, presence of lymphatic/vascular invasion, age at diagnosis, estrogen receptor status (ER), progesterone receptor status (PR), and HER-2 status have been previously published as independent variables for LN positivity [15–17]. Axillary lymph node involvement remains the most important prognostic factor in early-stage breast cancer. The observed higher frequency of node metastases in HER-2+ may account for the higher rates of local recurrence observed in HER-2-positive tumors. Nguyen et al. [3] recently studied 793 patients with invasive breast cancer of different molecular subtypes and treated with surgery and radiotherapy. The rate of local recurrence was 0.8 % for the luminal A subtype, 1.5 % for luminal B, 7.1 % for the TNBC group, and 8.4 % for the HER-2+ subtype. Even though there is no statistically significant difference among subtypes in lymph node metastases, our findings of increased nodal involvement in HER-2+ subtypes may at least partly explain the observed increase in local recurrence in the HER-2+ subtype in this study. Our data also support a previously reported study by Crabb et al. [18] that indicates that the TNBC subtype is associated with a lower incidence of axillary nodal involvement than other subtypes in four or more metastatic lymph nodes despite its poor prognosis. Meanwhile, it reported that the luminal B and HER-2+ subtypes did not predict a different risk of axillary lymph node involvement compared to the luminal A group. Our data support this finding, and we found that the HER-2+ subtype is associated with a higher likelihood of axillary metastases, despite not identifying a higher risk of having any positive nodes.

Even so, there are potential implications for the evaluation of patients for local therapy. In general, patients are fit for breast-conserving surgery with a high degree of accuracy by history, physical examination, and diagnostic mammography [19]. We found that patients overexpressing HER-2 (luminalHer2+ and HER-2+) and TNBC subtypes were more frequently in grade 3 and T3, and those overexpressing the HER-2+ subtype were more likely to have involvement of nodes, suggesting that these patients who are borderline candidates for breast conservation as a result of a large tumor size relative to the breast size would particularly likely benefit from neoadjuvant therapy that includes trastuzumab. This treatment has reported high pathologic complete response rates with the approach [20]. Discovery of nodal status before neoadjuvant therapy with axillary ultrasound and fine-needle aspiration may also be particularly profitable for the Her-2+ subtype with greater likelihood of involvement. This means most of these patients will benefit from chest wall irradiation when mastectomy is performed, and the time and cost of sentinel node biopsy could be saved even for someone who is not receiving neoadjuvant therapy.

Although the idea of using molecular subtype to select local therapy is appealing, we are not suggesting that the HER-2+ or luminalHer2+ subgroups be preferentially treated by mastectomy. Randomized trials have demonstrated that the addition of trastuzumab to chemotherapy decreases local failure by approximately 50 % compared with treatment with chemotherapy alone, so it is likely that rates of local failure in these groups have decreased [9]. In addition, Kyndi et al. [4] examined the risks of locoregional recurrence after mastectomy with and without radiotherapy based on the subtype classification, and they found higher rates of failure in HER-2+ than others. Collectively, these findings suggest that HER-2 status (in the absence of trastuzumab treatment) is a poor prognostic factor but is still not predictive of appropriate local therapy.

Our findings support those of Wiechmannet al. [21], showing that after controlling for patient age, tumor size, and grade, TNBC had an OR of 0.7 for one or more positive nodes and 0.8 for ≥4 metastatic lymph nodes. The relative likelihood of lymph node involvement, which is considered a poor prognostic indicator, is lower for TNBC compared to other breast cancer subtypes in four or more metastatic lymph nodes. It is suggested by this finding that the aggressive nature of TNBC is not directly associated with lymphatic spread, and it is consistent with the findings of other studies [7, 18, 21–24].

Tumor size was known as the most significant predictor of LN metastases [25, 26]. Similarly, our study demonstrated tumor size as a significant independent predictive factor for positive LN status with an odds ratio of 2.3 for T2 vs. T1 tumors and 2.7 for T3 vs. T1 tumors. In other studies, Mustafa et al. [27], when reviewing more than 2100 patients with ≤1 cm invasive tumors, show by multivariate analyses that not only the size of the tumor, but also the grade of the tumor and the patient’s age are related to the incidence of nodal involvement. However, we only found that in tumors of all sizes, higher grade was predictive of nodal positivity. Age is not associated with increased likelihood of nodal metastases [25, 26, 28].

## Conclusions

The results of our study are unexpected which suggest predictors of LN metastases only include higher grade and larger tumor size, and breast cancer subtype is not a statistically significant predictor of LN positivity. Even so, this information may still be useful in clinical practice. We found tumors overexpressing HER-2 (luminalHer2+ and HER-2+) and TNBC subtypes were more likely in grade 3 and T3 and HER-2+ tumors were associated more frequently with involved nodes. These are beneficial to make decisions regarding neoadjuvant therapy, breast-conserving therapy, axillary surgery, and locoregional radiation.
